# The relationship between the use of GLP-1 receptor agonists and the incidence of respiratory illness: a meta-analysis of randomized controlled trials

**DOI:** 10.1186/s13098-023-01118-6

**Published:** 2023-07-26

**Authors:** Meixin Yu, Ruxin Wang, Ling Pei, Xiaofang Zhang, Jinjing Wei, Yun Wen, Han Liu, Haowen Ye, Jinghao Wang, Lihong Wang

**Affiliations:** 1 Department of Endocrinology and Metabolism, The First Affiliated Hospital of Jinan University, No. 613, Huang pu Avenue West, Guangzhou, Guangdong China; 2 Clinical Experimental Center, The First Affiliated Hospital of Jinan University, No. 613, Huang pu Avenue West, Guangzhou, Guangdong China; 3 Department of Pharmacy, The First Affiliated Hospital of Jinan University, No. 613, Huang pu Avenue West, Guangzhou, Guangdong China; 4 The Guangzhou Key Laboratory of Basic and Translational Research on Chronic Diseases, The First Affiliated Hospital, Jinan University, Guangzhou, China

**Keywords:** GLP-1RA, Diabetes mellitus, type 2, Respiratory illness, Randomized controlled trial, Meta-analysis

## Abstract

**Aim:**

We aimed to assess the association between the use of Glucagon-like peptide-1 receptor agonists and the risk of 12 respiratory diseases in patients with type 2 diabetes, obesity, or overweight.

**Method:**

The PubMed (MEDLINE), EMBASE, Cochrane Library, and ClinicalTrials.gov databases were searched from the establishment of the database to December 24, 2022. Dichotomous outcomes were analyzed using RR and 95% CI calculated from fixed-effects models.

**Results:**

Twenty-eight RCTs were ultimately included for analysis, involving a total of 77,485 participants. Compared to controls, patients with GLP-1RAs have a 14% lower risk of respiratory disease (RR 0.86, 95% CI 0.81–0.93 p < 0.0001), with Semaglutid (RR 0.82, 95% CI 0.68–0.97, p = 0.02), Liraglutide (RR 0.86. 95% CI 0.75–0.98, p = 0.03), Dulaglutide (RR 0.82, 95% CI 0.70–0.96, p = 0.02), Albiglutide (RR 0.93,95% CI 0.79–1.10, p = 0.40), Exenatide (RR 0.93, 95% CI 0.74–1.18, p = 0.55), Lixisenatide (RR 0.83, 95% CI 0.62–1.12, p = 0.22), and Efpeglenatide (RR 0.76, 95% CI 0.46–1.24, p = 0.27). Semaglutide, Liraglutide and Dulaglutide reduce the risk of respiratory diseases by 18%, 14% and 18%, respectively.Trial duration, control type, and indication were not associated with the impact of GLP-1 receptor agonists on overall respiratory disease. Among secondary outcomes, the risk of Pulmonary edema (RR 0.66, 95% CI 0.44–0.98, p = 0.04), and Bronchitis (RR 0.86, 95% CI 0.74–1.00, p = 0.04) was reduced.

**Conclusion:**

In conclusion, GLP-1RAs were linked to a lower risk of overall respiratory diseases, especially Pulmonary edema and Bronchitis. In the future, physicians should pay attention to the relationship between GLP-1 RA and the risk of respiratory diseases and evaluate the efficacy of GLP-1RAs in the primary and secondary prevention of respiratory diseases.

*Trial registration* CRD42023396138.

**Graphical Abstract:**

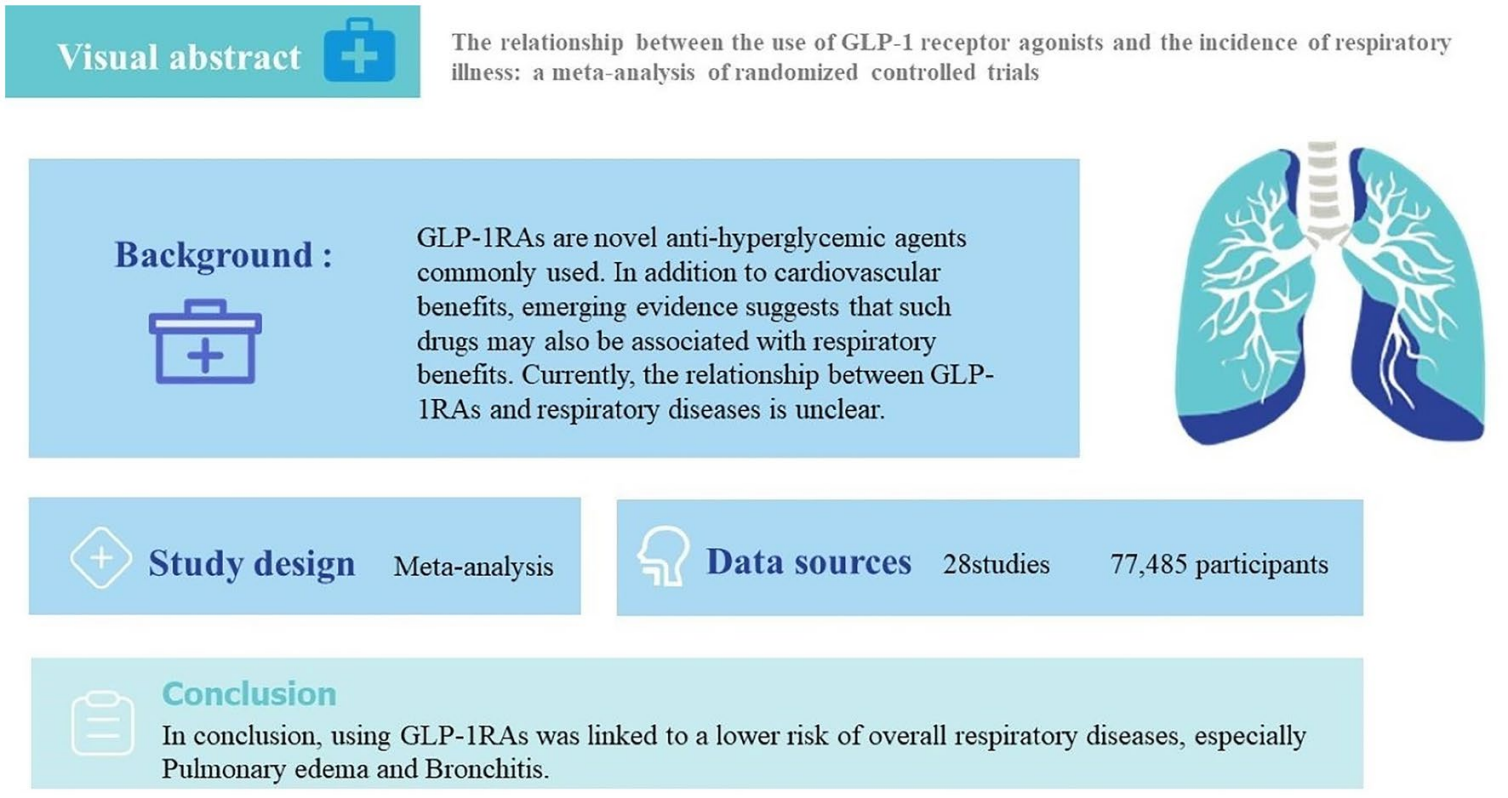

**Supplementary Information:**

The online version contains supplementary material available at 10.1186/s13098-023-01118-6.

## Introduction

Respiratory diseases are common disorders in life. Respiratory diseases are increasing in mortality and morbidity globally each year. It is a major contributor to the global health burden [1]. In addition, chronic respiratory disease is one of the top 10 causes of death worldwide [2]. Diabetes is a common comorbidity among patients with respiratory diseases. People with diabetes are at increased risk of developing sleep apnoea syndrome, chronic obstructive pulmonary disease (COPD), asthma, and lung injury. Hyperinsulinemia, hyperglycemia, and chronic pro-inflammatory states can lead to impaired lung function. There is evidence that GLP-1RAs may improve lung function through mechanisms such as reducing inflammation and tracheal hyperresponsiveness [3, 4].

GLP-1RAs are novel anti-hyperglycemic agents commonly used. In addition to cardiovascular benefits, emerging evidence suggests that such drugs may also be associated with respiratory benefits, for example in patients with COPD [5, 6].

Recently, a population-based cohort study (involving 56,243 participants) discovered that GLP-1 receptor agonists reduce the incidence of severe exacerbation of COPD [6]. Compared with sulfonylureas, GLP-1 receptor agonists were associated with a 30% decreased risk of severe exacerbation (3.5 v 5.0 events per 100 person years; HR 0.70, 95% CI 0.49–0.99) and moderate exacerbation (0.63, 0.43–0.94). Besides, in a previous retrospective cohort study, the use of GLP-1RA was found to result in fewer asthma attacks in adult asthmatics with type 2 diabetes [7]. GLP-1R agonists (reference) compared with SGLT-2 inhibitors (incidence rate ratio [IRR], 2.98; 95% confidence interval [CI], 1.30–6.80), DPP-4 inhibitors (IRR, 2.45; 95% CI 1.54–3.89), sulfonylureas (IRR, 1.83; 95% CI 1.20–2.77), and basal insulin (IRR, 2.58; 95% CI 1.72–3.88). Also, it allowed patients to potentially improve from obstructive sleep apnea syndrome and have less aggravation of the chronic lower respiratory disease [8, 9]. The adjusted incidence rate of first chronic lower respiratory disease admission during follow-up was 10.7 and 20.3 per 1000 person-years for GLP-1RA and DPP-4I users, respectively, resulting in an adjusted hazard ratio of 0.52 (95% CI 0.32–0.85). From the perspective of its possible underlying mechanism. The use of GLP-1RA was found to reduce inflammation and promote lung function in a mouse model of obstructive lung disease [10]. In another previous animal model, liraglutide pretreatment reduced acute pulmonary damage brought on by LPS in mice [11]. Furthermore, GLP-1RA has been shown to significantly attenuate LPS-stimulated eosinophil activation [12]. It may have immunomodulatory effects. Currently, the relationship between GLP-1RAs and respiratory diseases is unclear. To determine the link between GLP-1 agonists and the occurrence of respiratory diseases, we conducted a meta-analysis of all RCTs that satisfied the inclusion criteria. Meanwhile, since GLP-1RAs are clinically used for type 2 diabetes and weight loss, and diabetes and obesity are risk factors for the development of respiratory diseases, we included people with type 2 diabetes, obesity, or overweight for the study [5, 13].

## Methods

### Data sources and searches

We searched PubMed (MEDLINE), EMBASE, and Cochrane Library databases to find literature on GLP-1RA published from database creation to December 24, 2022, and the literature was not restricted by language. The following were the search keywords used: “Glucagon-like peptide 1 receptor agonist”, “liraglutide”, “albiglutide”, “lixisenatide”, “exenatide”, “dulaglutide”, “semaglutide”, “Diabetes Mellitus, Type 2”, “obesity”, “Overweight” and “randomized controlled trial”. Detailed search strategies can be found in the Additional file.

This meta-analysis follows the Preferred Reporting Items for Systematic Reviews and Meta-Analyses (PRISMA) reporting guidelines statement. The International Registry of Prospective Systematic Reviews has prospectively recorded the meta-procedure. (CRD42023396138).

### Study selection

Inclusion criteria: (1) Adult people with type 2 diabetes mellitus, overweight or obese. (2) RCT comparing GLP-1 receptor agonist with placebo or positive control (Insulin or other oral hypoglycemic drugs). (3) The results reported at least one respiratory disease event of interest (all data from ClinicalTrials.gov on respiratory adverse events).

Exclusion criteria: (1) Pregnant women. (2) GLP-1RA in combination with other hypoglycemic agents (e.g. IDegLira). (3) Comparison between different GLP-1RAs (4) Multiple publications from the same RCT (using the most complete or most recently reported data), reviews, case reports, letters, and conference proceedings on GLP-1RA. (4) Studies with incomplete data or full text not available (Table 1).

**Table 1:** Inclusion and exclusion criteria

	Inclusion criteria	Exclusion criteria
Participants	Adults(≥ 18 years old) with type 2 diabetes, obesity, or overweight	Pregnant women
Intervention	Use any GLP-1RA, GLP-1RA can also be used as add-on therapy to other interventions	Combination drugs of GLP-1RA or combination with other hypoglycemic drugs
Comparators	Placebo or active control (other antidiabetic drugs or insulin)	Comparing different categories of GLP-1RAs
Outcomes	Respiratory diseases	No relevant outcome data is available
Study design	RCTs	Post-hoc analysis, multiple articles from the same study

### Outcome measures

Primary outcome: overall respiratory disease prevalence.

Secondary outcomes: 12 subcategories of respiratory diseases: Pulmonary edema , pneumonia, Bronchitis, Pulmonary fibrosis, Dyspnoea, Acute respiratory failure, Pleural effusion, Asthma, COPD, Sleep apnoea syndrome, Pulmonary embolism, Pulmonary hypertension.

### Data extraction and quality assessment

By reading the full text of the included literature, two investigators (MX and RX) independently extracted the following data: First author, year of study publication, clinical trial registration number, duration of follow-up, type of GLP-1 RA, control drug, and trial subject characteristics (age, sex, BMI, and glycosylated hemoglobin [HbA1c] levels). Any discrepancies arising from this process were resolved by consensus or by a third reviewer (PL). The Cochrane Risk of Bias tool was used to assess the quality of each RCT included [14].

### Statistical analysis

We analyzed dichotomous outcomes using RR and 95% CI calculated from random or fixed effects models. Heterogeneity between studies was assessed using the χ2 test and the I^2^ statistic. If heterogeneity was low (P > 0 0.05; I^2^ ≤ 50%), analyses were performed using the Mantel–Haenszel method and fixed-effects models. Otherwise, a random-effects model using the DerSimonian-Laird method was used. We also assessed the association of specific GLP-1 RA drugs with respiratory disease. Also, we performed subgroup analyses according to control type, trial duration, and indication. Sensitivity analyses were performed by omitting eligible trials one by one. We used a funnel plot and the Egger test to check for publication bias. All of the analyses were conducted using RevMan 5.4 and Stata 14. We think it is statistically significant if the P-value is < 0.05.

## Result

### Study search and study characteristics

**Fig. 1:**
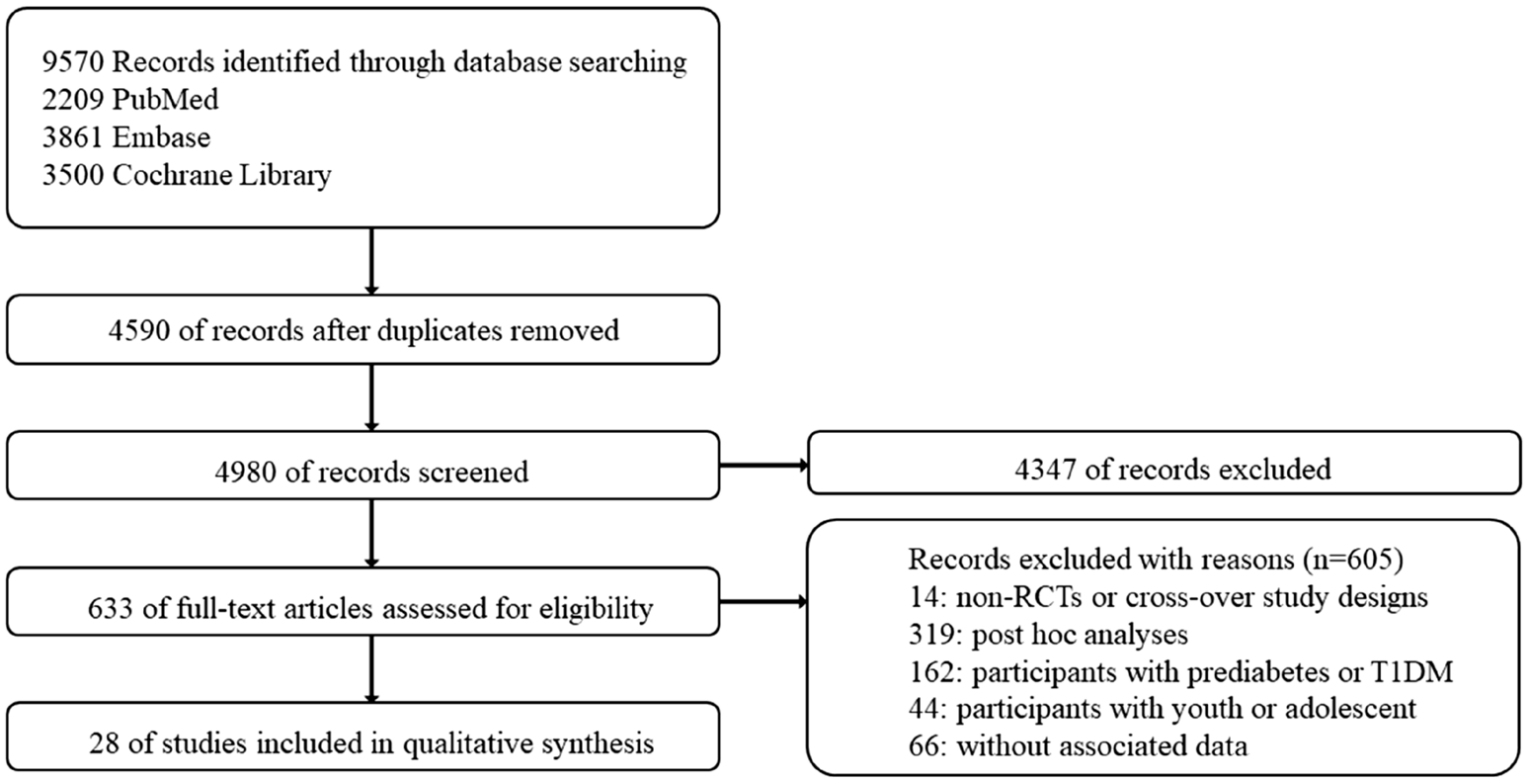
Process of trial selection

A search of the above databases based on keywords returned 9570 records in total. After excluding duplicates, and reading the titles and abstracts, a total of 633 documents were read in full. A total of 28 RCTs involving a total of 77485 participants were finally included for analysis [15–42]. Figure 1 shows the study screening flow chart. In the 28 included trials, the majority included subjects with type 2 diabetes (96.3%) with follow-up periods ranging from 24 weeks to 5.4 years. Among all participants, the average age of the study participants was between 46.0 and 74.2 years. The proportion of males ranged from 19.0 to 69.4%. The average BMI varied from 26.8 to 38.5 kg/m^2^. Mean glycosylated hemoglobin (HbA1c) ranged from 5.7 to 8.9%. (Table 2).

**Table 2:** Baseline characteristics of included studies

Study	NCT ID	Country/Region	Indication	Type of underlying diseases	Experimental	Control	Trial duration	Total	Average age(years)	Male (n,%)	BMI(kg/m2)	Mean HbA1c (%)
									Mean (Standard Deviation)		Mean (Standard deviation)	Mean (Standard deviation)
LEAD -2 (2009)	NCT00318461	Europe, Oceania, Africa, Asia and South America	T2DM	T2DM	liraglutide 0.6 , 1.2, 1.8 mg once daily	placebo, glimepiride	26 week	1091	56.7 (9.5)	58.20	31.0 (4.7)	8.4 (0.9)
LIRA-RENAL(2016)	NCT01620489	France, Poland, Russian Federation, Ukraine, United Kingdom, United States	T2DM	T2DM and moderate renal impairment	liraglutide 1.8 mg once daily	Placebo	26 week	279	67.2 (8.2)	50.50	33.9 (5.4)	8.0 (0.8)
HARMONY 3(2014)	NCT00838903	Albania, Germany, Hong Kong, Mexico, Peru, Philippines, Russian Federation, South Africa, Spain, United Kingdom, United States	T2DM	T2DM	Albiglutide 30 mg once weekly	Placebo, Sitagliptin, Glimepiride	104 week	1012	54.5 (8.0)	47.60	32.6 (5.5)	8.1 (0.8)
Gallwitz et al. (2011)	NCT00434954	Germany	T2DM	T2DM	Exenatide 10 μg twice daily	Premixed Insulin Aspart	26 week	494	57.1 (10.0)	57.60	33.2 (4.3)	7.88 (0.9)
GetGoal-O (2017)	NCT01798706	Australia, Bulgaria, Canada, Denmark, Germany, Norway, Peru, Poland, South Africa, Spain, Sweden, United Kingdom, United States	T2DM	T2DM	Lixisenatide 20 μg once daily	Placebo	24 week	350	74.2 (3.9)	52.00	30.0 (4.1)	8.0 (0.7)
AWARD-CHN2 (2019)	NCT01648582	China, Mexico, and Russian Federation	T2DM	T2DM	Dulaglutide 0.75, 1.5 mg once weekly	Insulin Glargine	52 week	774	55.0 (9.6)	55.20	26.8 (3.7)	8.4 (1.1)
AWARD-5 (2015)	NCT00734474	United States, Canada, France, Germany,India, Korea, Mexico, Poland, Puerto Rico,Romania, Russian Federation, Spain, Taiwan	T2DM	T2DM	Dulaglutide 0.25, 0.5, 0.75, 1.0, 1.5, 2.0, 3.0 mg once weekly	sitagliptin	104 week	921	54.1 (9.9)	46.50	31.3 (4.4)	8.1 (1.1)
HARMONY 4 (2014)	NCT00838916	United States, Russian Federation, South Africa, United Kingdom	T2DM	T2DM	Albiglutide 30 mg once weekly	insulin glargine	52 week	745	55.5 (9.5)	56.10	33.1(5.5)	8.3 (0.9)
Charbonnel et al. (2013)	NCT01296412	Canada, Colombia, Czech Republic, Denmark, Finland, France, Germany, Hong Kong, Hungary, Israel, Italy, Lithuania, New Zealand, Poland, Puerto Rico, Slovakia, Slovenia, Spain, Sweden, United Kingdom, United States	T2DM	T2DM	liraglutide 1.2 mg once daily	sitagliptin	26 week	653	57.3 (10.4)	54.80	32.7(6.0)	8.2 (1.0)
SCALE Diabetes (2015)	NCT01272232	Argentina, Brazil, France, Germany, India, Israel, Japan, Mexico, Romania, Russian Federation, South Africa, Turkey, Ukraine, United Kingdom, United States	Weight Loss	T2DM and overweight or obesity	Liraglutide 1.8, 3.0 mg once daily	Placebo	56 week	846	54.9 (10.5)	50.20	37.1 (6.8)	7.9 (0.8)
PIONEER 3 (2019)	NCT02607865	France, Germany, India, Israel, Italy, Puerto Rico, South Africa, Spain, Sweden, Taiwan, Turkey, United Kingdom, United States	Cardiovascular Outcomes	T2DM	Oral Semaglutide 3, 7, 14 mg once daily	Sitagliptin	78 week	1864	58.0 (10.0)	52.80	32.5(6.4)	8.3 (0.9)
STEP 3 (2021)	NCT03611582	United States	Weight Loss	Overweight or Obesity (without diabetes)	Semaglutide 2.4 mg once weekly	Placebo	68 week	611	46.0 (13.0)	19.00	38.0(6.7)	5.7 (0.3)
Harmony Outlets (2018)	NCT02465515	Argentina, Belgium, Bulgaria, Canada, Czechia, Denmark, France, Germany, Greece, Hong Kong, Hungary, Italy, Korea, Republic of, Mexico, Netherlands, Norway, Peru, Philippines, Poland, Russian Federation, South Africa, Spain, Sweden, Taiwan, Thailand, Ukraine, United Kingdom, United States	Cardiovascular Outcomes	T2DM and cardiovascular disease	Albiglutide 30, 50 mg once weekly	Placebo	1.6 Year	9463	64.1 (8.7)	69.40	32.3(5.9)	8.7(1.5)
STEP 2 (2021)	NCT03552757	Argentina, Canada, Germany, Greece, India, Japan, Puerto Rico, Russian Federation, South Africa, Spain, United Arab Emirates, United Kingdom, United States	Weight Loss	T2DM and overweight or obesity	Semaglutide 1.0, 2.4 mg once weekly	Placebo	68 week	1210	55.0 (11.0)	49.10	35·7 (6·3)	8·1 (0·8)
REWIND (2019)	NCT01394952	Argentina, Australia, Brazil, Bulgaria, Canada, Chile, Colombia, Czechia, Germany, Hungary, Korea, Republic of, Latvia, Lithuania, Mexico, New Zealand, Poland, Puerto Rico, Romania, Russian Federation, South Africa, Spain, Sweden, Taiwan, United Kingdom, United States	Cardiovascular Outcomes	T2DM and cardiovascular disease( or high cardiovascular risk)	Dulaglutide 1.5 mg once daily	Placebo	5.4 Year	9901	66.2 (6.5)	53.65	32.3 (5.7)	7.4 (1.1)
AWARD-4 (2015)	NCT01191268	Argentina, Australia, Belgium, Brazil, Canada, Denmark, Greece, Hungary, Mexico, Poland, Puerto Rico, Russian Federation, Spain, Sweden, Taiwan, United States	T2DM	T2DM	Dulaglutide 0.75, 1.5 mg once weekly	insulin glargine	52 week	884	59.4 (9.2)	53.50	32.5 (5.2)	8.5 (1.1)
EUREXA (2012)	NCT00359762	Austria, Czech Republic, Finland, France, Germany, Hungary, Ireland, Israel, Italy, Mexico, Poland, Spain, Switzerland, United Kingdom	T2DM	T2DM	Exenatide 10 μg twice daily	glimepiride	2.0 Year	1029	56.4 (9.6)	53.60	32.5 (4.1)	7.4 (0.70)
LEAD-3 Mono (2009)	NCT00294723	Mexico, Puerto Rico, United States	T2DM	T2DM	liraglutide 1.2 , 1.8 mg once daily	glimepiride	104 week	746	53.0 (10.9)	49.70	33.1 (5.8)	8.3 (1.1)
SUSTAIN 2 (2017)	NCT01930188	Argentina, Bulgaria, Czechia, Hong Kong, Hungary, India, Japan, Mexico, Norway, Portugal, Romania, Russian Federation, South Africa, Spain, Sweden, Thailand, Turkey, Ukraine	T2DM	T2DM	Semaglutide 0.5, 1.0 mg once weekly	Sitagliptin	56 week	1225	55.1 (10.0)	50.60	32.5(6.2)	8.07 (0.9)
LEADER (2016)	NCT01179048	Australia, Austria, Belgium, Brazil, Canada, China, Czechia, Denmark, Finland, France, Germany, Greece, India, Ireland, Israel, Italy, Korea, Republic of, Mexico, Netherlands, Norway, Poland, Puerto Rico, Romania, Russian Federation, Serbia, South Africa, Spain, Sweden, Taiwan, Turkey, United Arab Emirates, United Kingdom, United States	Cardiovascular Outcomes	T2DM and high cardiovascular risk	liraglutide 1.8 mg once daily	Placebo	3.8 Year	9340	64.3 (7.2)	64.30	32.5 (6.3)	8.7 (1.6)
PIONEER 6 (2019)	NCT02692716	Algeria, Argentina, Brazil, Canada, Denmark, Germany, India, Israel, Italy, Malaysia, Mexico, Netherlands, Poland, Romania, South Africa, Spain, Taiwan, Thailand, Turkey, United Kingdom, United States	Cardiovascular Outcomes	T2DM and high cardiovascular risk	Oral Semaglutide 14 mg once daily	Placebo	1.3 Year	3183	66.0 (7.0)	68.40	32.3 (6.5)	8.2 (1.6)
ELIXA (2015)	NCT01147250	Argentina, Australia, Austria, Belarus, Belgium, Brazil, Bulgaria, Canada, Chile, China, Colombia, Denmark, Ecuador, Egypt, Estonia, Finland, France, Georgia, Germany, Guatemala, India, Israel, Italy, Japan, Korea, Republic of, Latvia, Lithuania, Mexico, Netherlands, Norway, Panama, Peru, Philippines, Poland, Portugal, Romania, Russian Federation, Serbia, South Africa, Spain, Sweden, Switzerland, Taiwan, Tunisia, Turkey, Ukraine, United Arab Emirates, United Kingdom, United States	Cardiovascular Outcomes	T2DM and Acute Coronary Syndrome	Lixisenatide 20 μg once daily	Placebo	2.1 Year	6068	60.3 (9.7)	69.30	30.2 (5.7)	7.7 (1.3)
EXSCEL (2017)	NCT01144338	Argentina, Australia, Austria, Belgium, Brazil, Bulgaria, Canada, Chile, China, Colombia, Czechia, Germany, Hong Kong, Hungary, Israel, Italy, Korea, Republic of, Latvia, Lithuania, Malaysia, Mexico, Netherlands, New Zealand, Philippines, Poland, Romania, Russian Federation, Slovakia, South Africa, Spain, Taiwan, Thailand, Ukraine, United Kingdom, United States	Cardiovascular Outcomes	T2DM	Exenatide 2 mg once weekly	Placebo	3.2 Year	14,752	61.9 (9.4)	62.00	31.7 (5.9)	8.0 (1.2)
Wilding et al. (2021)	NCT03548935	Argentina, Belgium, Bulgaria, Canada, Denmark, Finland, France, Germany, India, Japan, Mexico, Poland, Puerto Rico, Russian Federation, Taiwan, United Kingdom, United States	Weight Loss	Overweight or Obesity (without diabetes)	Semaglutide 2.4 mg once weekly	Placebo	68 week	1961	46.0 (13)	25.90	37.9 (6.6)	5.7 (0.3)
SUSTAIN-6 (2016)	NCT01720446	Algeria, Argentina, Australia, Brazil, Bulgaria, Canada, Denmark, Germany, India, Israel, Italy, Malaysia, Mexico, Poland, Russian Federation, Spain, Taiwan, Thailand, Turkey, United Kingdom, United States	Cardiovascular Outcomes	T2DM and cardiovascular disease	Semaglutide 0.5, 1.0 mg once weekly	Placebo	2.1 Year	3297	64.6 (7.4)	60.70	32.8 (6.2)	8.7 (1.5)
STEP 5 (2022)	NCT03693430	Canada, Hungary, Italy, Spain, United States	Weight Loss	Overweight or Obesity (without diabetes)	Semaglutide 2.4 mg once weekly	Placebo	104 week	304	47.0 (11.0)	22.40	38.5 (6.9)	5.7 (0.4)
AMPLITUDE-O (2021)	NCT03496298	Argentina, Bulgaria, Canada, Chile, Denmark, Estonia, Finland, Germany, Hungary, India, Italy, Korea, Republic of, Latvia, Lithuania, Mexico, Norway, Peru, Poland, Romania, Russian Federation, Serbia, Slovakia, South Africa, Spain, Sweden, Turkey, Ukraine, United States	Cardiovascular Outcomes	T2DM and cardiovascular disease or kidney disease	Efpeglenatide 4,, 6 mg once weekly	Placebo	1.81 years	4076	64.5 (8.2)	67	32.7 (6.2)	8.9(1.5)
AMPLITUDE-M(2022)	NCT03353350	Germany, Poland, Ukraine, United Kingdom, United States	T2DM	T2DM	Efpeglenatide 2, 4, 6 mg once weekly	Placebo	56 week	406	58.5 (11.2)	53.90	34.2 (6.8)	8.1 (0.9)

### Risk of bias evaluation

**Fig. 2:**
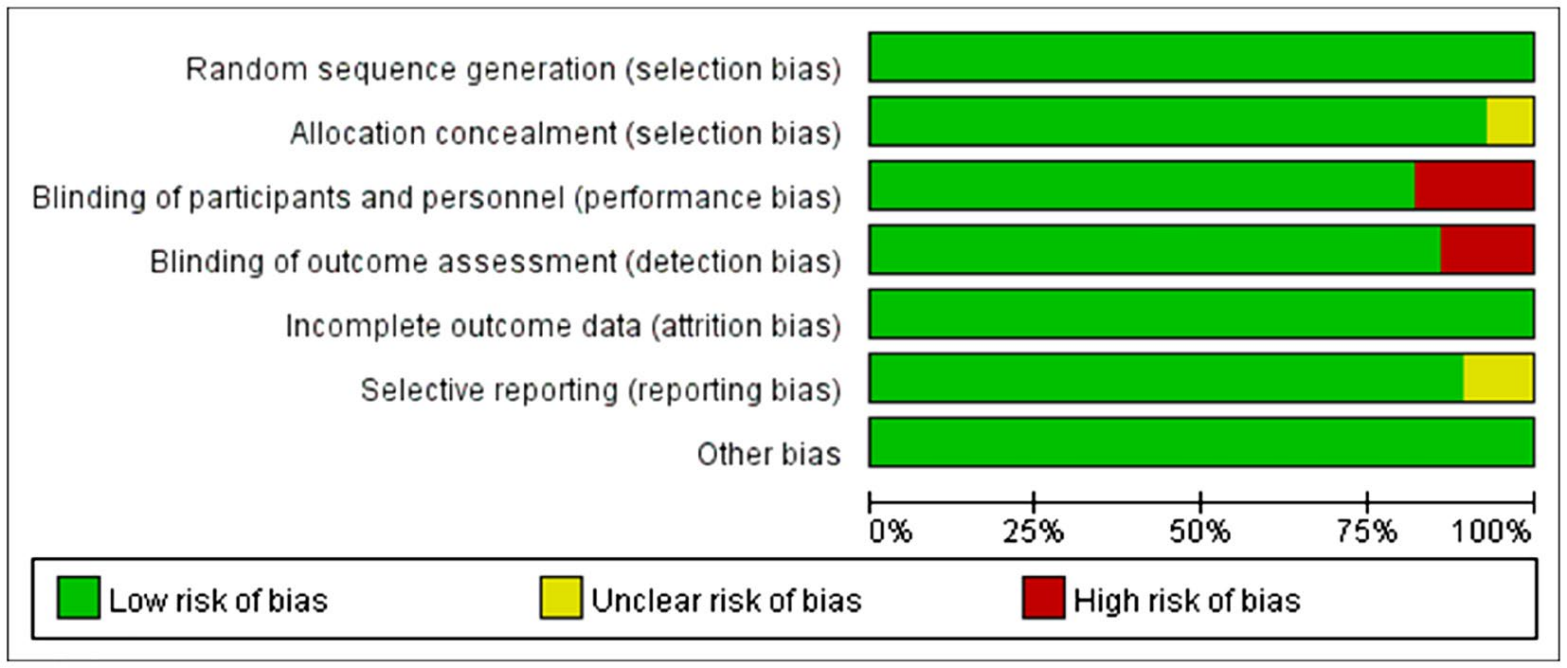
Quality assessment (Cochrane risk of bias tool) for included RCTs

The Cochrane Risk of Bias tool, which provides information on the risk of bias evaluation, was used to evaluate the quality of the included studies (Fig. 2).

### Main outcome

#### Incidence of respiratory diseases with all GLP-1 receptor agonists

**Fig. 3:**
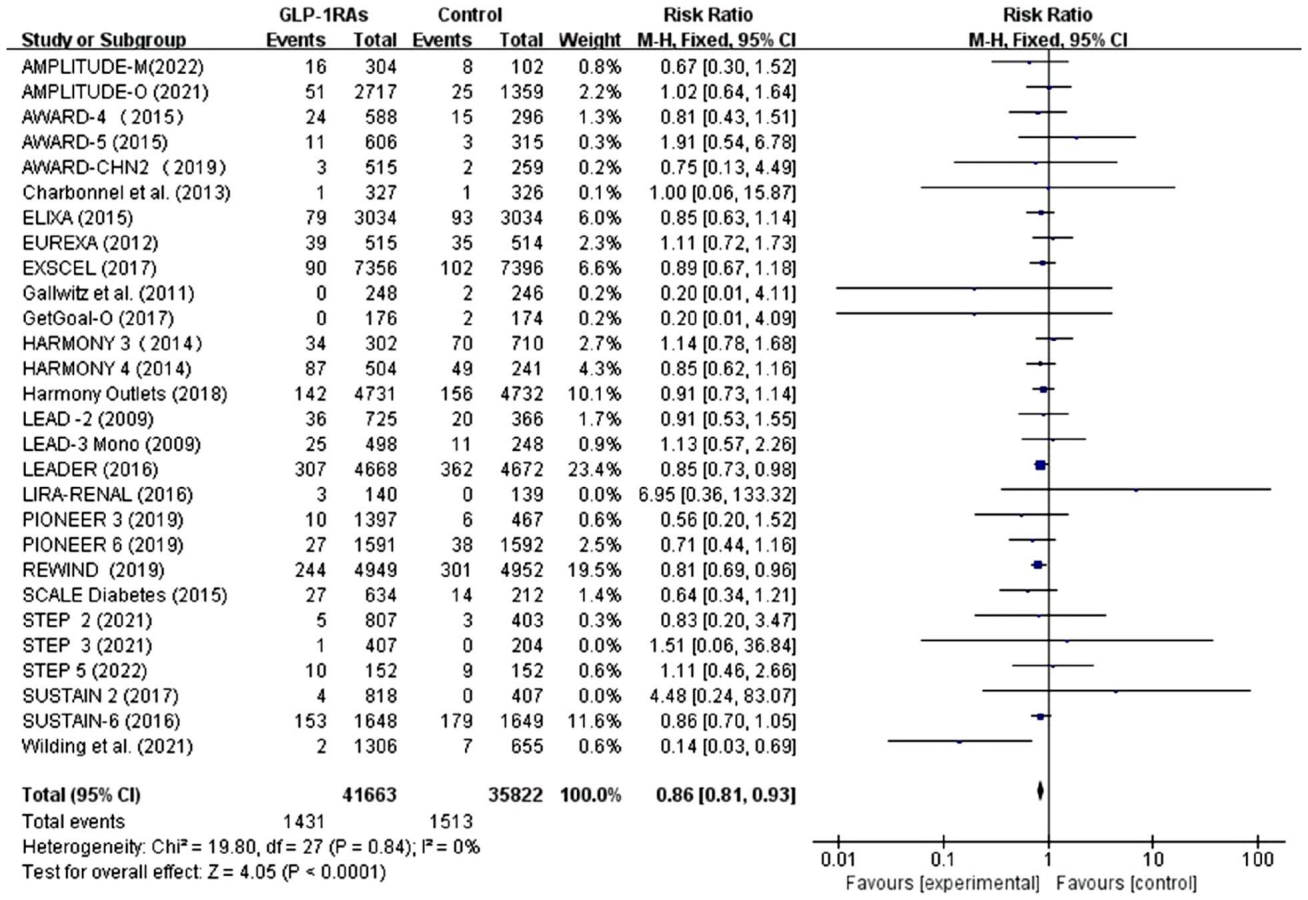
Forest plot of GLP-1 receptor agonists versus comparators on the risk of overall Respiratory diseases

As shown in Fig. 3, this study included 41,663 participants using GLP-1RAs and 35,822 patients using placebo, oral hypoglycemic agents, or insulin. The GLP-1 RAs group had a lower event rate (3.43%) than the control group (4.22%). Patients with GLP-1RAs had a 14% lower risk of respiratory disease compared to controls (RR 0.86, 95% CI 0.81–0.93; p < 0.0001), with no significant heterogeneity between studies (I^2^ = 0). In the sensitivity analysis, the pooled results were not significantly altered after repeated omission of each trial. There was no obvious publication bias, according to the funnel plots of this analysis. (Additional file 1: Figure S1, Egger’s test P = 0.859 > 0.05).

#### Incidence of respiratory diseases with different GLP-1 receptor agonists

**Fig. 4:**
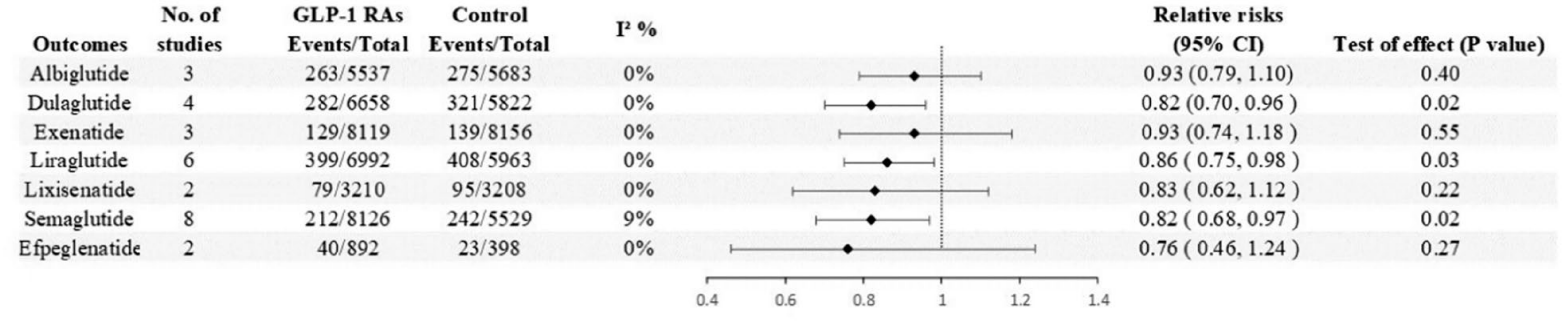
Forest plot of specific GLP-1 receptor agonists versus comparators on the risk of overall Respiratory diseases

As shown in Fig. 4, eight trials totaling 13655 patients were included with Semaglutide as the experimental group. Semaglutide reduced the risk of developing respiratory diseases by 18% compared to placebo or other interventions (RR 0.82, 95% CI 0.68–0.97, p = 0.02). In addition, six trials totaling 12,955 patients were included with Liraglutide as the experimental group. Compared to the control group, Liraglutide reduced the risk of respiratory disease by 14% (RR 0.86, 95% CI 0.75–0.98, p = 0.03). Four RCT studies including 12,480 patients provided information on the use of Dulaglutide and the risk of respiratory disease. The results showed that Dulaglutide reduced the risk of respiratory disease by 18% compared to controls (RR 0.82, 95% CI 0.70–0.96, p = 0.02).

However, other GLP-1 receptor agonists did not affect the overall respiratory disease. The RR for the occurrence of respiratory disease in patients receiving Albiglutide was 0.93 (95% CI 0.79–1.10, p = 0.40) compared to other interventions. The RR for the occurrence of respiratory disease in patients receiving Exenatide was 0.93 (95% CI 0.74–1.18, p = 0.55). The RR for the occurrence of respiratory disease in patients receiving Lixisenatide was 0.83 (95% CI 0.62–1.12, p = 0.22). The RR for the occurrence of respiratory disease in patients receiving Efpeglenatide was 0.76 (95% CI 0.46–1.24, p = 0.27). No inter-study heterogeneity was observed in all of the above analyses.

#### Subgroup analyses

**Fig. 5:**
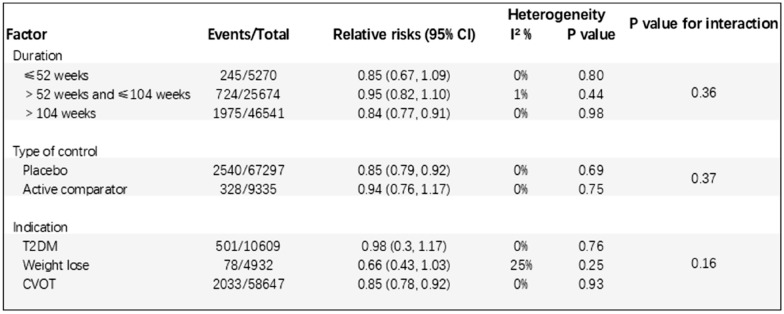
Subgroup analysis: GLP-1 receptor agonists and Respiratory disease. CVOT: Cardiovascular Outcomes Trial

Based on subgroup analysis of trial duration, control type, and indication. The results showed that trial duration, control type, and indication had no significant effect on the effect of GLP-1 receptor agonists on overall respiratory disease (all P subgroups > 0.05; Fig. 5).

### Secondary outcome

Random assignment to GLP-1 RA treatment resulted in a reduced risk of Pulmonary edema (RR 0.66, 95% CI 0.44–0.98, p = 0.04; I^2^ = 11; Additional file 1: Fig S3), Bronchitis (RR 0.86, 95% CI 0.74–1.00, p = 0.04; I^2^ = 0; Additional file 1: Fig S4). Meanwhile, GLP-1RAs showed a trend towards a reduced risk for nine respiratory diseases, although not reaching statistical significance, including pneumonia (RR 0.89, 95% CI 0.78–1.00, p = 0.05; I^2^ = 0; Additional file 1: Fig S5), Pulmonary fibrosis (RR 0.39, 95% CI 0.13–1.16, p = 0.09; I^2^ = 0; Additional file 1: Fig. S6), Dyspnoea (RR 0.78, 95% CI 0.58–1.04, p = 0.09; I^2^ = 0; Additional file 1: Fig. S7), Acute respiratory failure (RR 0.81, 95% CI 0.57–1.13, p = 0.21; I^2^ = 0; Additional file 1: Fig. S8), Pleural effusion (RR 0.73, 95% CI 0.48–1.11, p = 0.15; I^2^ = 6; Additional file 1: Figure S9), Asthma (RR 0.85, 95% CI 0.59–1.24, p = 0.40; I^2^ = 0; Fig S10), and COPD (RR 0.93. 95% CI 0.76–1.13, p = 0.47; I^2^ = 0; Additional file 1: Fig S11), Sleep apnoea syndrome (RR 0.81, 95% CI 0.48–1.36, p = 0.43; I^2^ = 0; Additional file 1: Fig S12), Pulmonary embolism (RR 0.95, 95% CI 0.69–1.31, p = 0.76; I^2^ = 0; Additional file 1: Fig S13). GLP-1RAs did not affect Pulmonary hypertension (RR 1.02, 95% CI 0.51–2.05, p = 0.95; I^2^ = 5; Additional file 1: Fig S14). Heterogeneity was absent or low for all meta-analyses analyzed above.

## Discussion

The relationship between GLP-1RA and respiratory disease has rarely been studied in clinical studies. In 2021, the results of a meta-analysis that included 7 RCT studies showed that GLP-RA uses resulted in a decreased risk of respiratory disease [43]. This meta-analysis included large RCTs with cardiovascular or renal outcomes as experimental endpoints in patients with type 2 diabetes. Our meta-analysis mainly focuses on people with type 2 diabetes, obesity, or overweight. We expand the crowd even further. Furthermore, it is to determine whether the risk may differ by GLP-1RA type, trial duration, control type, and indication. In this regard, we have further discussed and systematically elaborated.

This meta-analysis examined the connection between the injection of GLP-1 receptor agonists and the incidence of diverse respiratory diseases. Two main discoveries were discovered. Firstly, compared to placebo or other therapies, GLP-1 RAs greatly decreased the incidence of total respiratory illness by 14%, particularly with Semaglutid, Liraglutide, and Dulaglutide. In the forest plot, several studies including Harmony Outlets (2018), LEADER (2016), REWIND (2019), and SUSTAIN-6 (2016) appear to show a low risk of respiratory diseases. These studies are all Cardiovascular Outcomes Trials, which are large multi-country and multi-center RCTs. The weight of these studies is between 10.1 and 23.4%, and the total number of participants is between 3297 and 9901. Median follow-up time of these studies ranged from 1.6 to 5.4 years. Therefore, more participants and longer follow-up time may better reflect the relationship between the use of GLP-1RAs and the risk of respiratory disease. It is meaningful to further evaluate the relationship between long-term use of GLP-1RAs and the risk of respiratory diseases in the future. In the secondary outcome, the risk of pulmonary edema and bronchitis was reduced. On the other hand, although statistical significance was not reached, the use of GLP-1RAs showed a trend towards a reduced risk of nine respiratory diseases (pneumonia, pulmonary fibrosis, dyspnea, acute respiratory failure, pleural effusion, COPD, sleep apnea syndrome, and pulmonary embolism).

GLP-1RAs were highly expressed in the lung [4]. Among the extra-pancreatic organs, the lung had the highest level of Glp1r mRNA expression [44, 45]. Moreover, alveolar lining fluid had GLP-1 concentrations that were substantially higher than those in serum [46]. This provides a pathway for a potential effect of GLP1-RA beyond glucose regulation. So far, the mechanisms underlying the protective effects of GLP-1RA in respiratory disease are not fully understood. GLP-1 RA may reduce tracheal hyperresponsiveness by activating the cAMP-dependent protein kinase A pathway in the human airways [4]. It also mediates tracheal relaxation. In a study using female mice in an obstructive pulmonary disease model, the use of GLP-1RA was found to reduce inflammation and improve lung function [10]. Meanwhile, some studies on GLP-1RA's anti-inflammatory effects in T2D patients have been conducted. GLP-1RA inhibits oxidative stress and inflammatory mediator production in patients with type 2 diabetes [47]. GLP-1RA dramatically lowered the levels of eosinophil surface activation markers after LPS stimulation. Thus, it attenuated LPS-stimulated eosinophil activation and reduced the generation of IL-4, IL-8, and IL-13 [12]. This suggests that GLP-1 may have an immunomodulatory role in asthma. In addition, in a previous animal model, liraglutide pretreatment attenuated LPS-induced acute lung injury in mice. It was linked to a decrease in the activity of inflammatory cytokines and chemokine genes in the lung, and it significantly decreased lung injury scores, and lung apoptosis [11]. In a related study, liraglutide was shown to increase FVC, and carbon monoxide diffusion capacity, and significantly reduce serum surface active protein D (SP-D) levels (used as a biomarker to assess alveolar-capillary barrier integrity) in patients with type 2 diabetes [48, 49]. It is well known that pulmonary surface-active substances (PS) are composed of various lipids and proteins arranged on the alveolar surface. Its main function is to maintain lung compliance and lung fluid homeostasis. on the other hand, surfactant protein A is the most prevalent protein in PS. As well, it has been shown that liraglutide increases the expression of SP-A by activating the TTF-1 signaling pathway. Thus, it may reduce the occurrence of pulmonary edema [50].

In the results of a cohort study published in BMJ, new users of GLP-1 receptor agonists had a 30% lower risk of severe COPD exacerbation compared to new sulfonylurea users. There was also a reduced risk of moderate exacerbation [6]. In another retrospective study, it was shown that patients starting GLP-1RA in intensive glucose-lowering therapy for patients with combined asthma and type 2 diabetes had fewer asthma attacks than those using other alternatives. Asthma symptoms were also reduced [7]. Interestingly, insulin resistance may enhance the pro-asthmatic effects of obesity. GLP-1 RAs may reduce asthma through weight loss and improved insulin sensitivity [51–53]. In addition, in a study that included 16,690 patients, patients with GLP-1RA experienced less worsening of chronic lower respiratory disease compared to DPP-4I users [8]. Finally, GLP-1 receptor agonists may improve lung function by reducing body weight, as abdominal obesity decreases lung capacity by pushing the diaphragm into the chest cavity.

In our analysis, a trend towards a decreased risk of sleep apnea syndrome was found with the use of GLP-1RA (RR 0.86, 95% CI 0.50–1.45). GLP-1 RAs may benefit patients with this obstructive sleep apnea syndrome by weight loss, reducing systemic inflammation, improving metabolic dysregulation, and reversing endothelial dysfunction [9]. Glucagon-like peptide-1 agonists have been advocated in the literature as a one-stop shop for the obesity-diabetes-OSA triad [9]. Besides, our results suggest that GLP-1RAs may not affect Pulmonary hypertension. However, in previous studies, it was demonstrated that GLP-1RAs could diastole pulmonary arteries in a cAMP-dependent manner [54]. Meanwhile, Glucagon-like peptide 1 receptor agonists attenuate autophagy via Drp1/NOX- and Atg-5/Atg-7/Beclin-1/LC3β pathways to improve pulmonary hypertension [55]. The inconsistent results may be due to the low incidence and the incorporation of studies with limited sample sizes, statistical effectiveness was not greatly increased. And it may lead to small sample effect bias. In the study of several categories of GLP-1 receptor agonists, we discovered that Liraglutide, Semaglutid, and Dulaglutide were connected to the low incidence of respiratory disease. This may be related to the high amino acid homology of natural glucagon-like peptide-1, with Liraglutide reaching 97% [56]. In addition, the chemical structures of different GLP-1 RAs with different pharmacological characteristics from each other. And these different findings could also be explained by unbalanced sample sizes.

This article has several advantages. Firstly, this is the first meta-analysis to evaluate the relationship between GLP-1RAs and the risk of respiratory disease in greater detail. The overweight or obese population was also included and subgroup analyses were carried out. In addition, all included studies were randomized controlled trials. Finally, there was either no heterogeneity or very little heterogeneity in our meta-analysis. We admit several limitations of our research. Almost all included studies did not have respiratory diseases as a primary outcome, and these outcomes were derived from adverse event reports while not detecting changes in lung function. Second, the analysis included only trials reporting respiratory disease, resulting in an unknown publication bias. In addition, although 28 studies were incorporated into this analysis, the wide confidence intervals reduced the quality of our findings. Finally, the limited amount of events in the subgroups may have led to a lack of certainty in the subgroup analysis. The effect of using GLP-1RAs on respiratory disease is unknown. More large long-term RCTs with respiratory disease as the primary outcome are needed in the future to evaluate the link between the incidence of respiratory disease and GLP-1 receptor agonists.

## Conclusion

In conclusion, using GLP-1RAs was linked to a lower risk of overall respiratory diseases, especially Pulmonary edema and Bronchitis. In the future, physicians should pay attention to the relationship between GLP-1 RA use and the risk of respiratory diseases and evaluate the efficacy of GLP-1RAs in the primary and secondary prevention of respiratory diseases.

## Supplementary Information

**Additional file 1: Figure S1.** Risk of bias summary. **Figure S2.** Funnel plot and Egger’s test for the comparison of the incidence of overall Respiratory diseases with the use of GLP-1 receptor agonists versus placebo or other antidiabetic treatments. **Figure S3.** Forest plot of GLP-1 receptor agonists versus comparators on the risk of Pulmonary edema. GLP-1RAs: GLP-1 receptor agonists, RR: risk ratios, CI: confidence Interval. **Figure S4.** Forest plot of GLP-1 receptor agonists versus comparators on the risk of Bronchitis. GLP-1RAs: GLP-1 receptor agonists, RR: risk ratios, CI: confidence Interval. **Figure S5.** Forest plot of GLP-1 receptor agonists versus comparators on the risk of pneumonia. GLP-1RAs: GLP-1 receptor agonists, RR: risk ratios, CI: confidence Interval. **Figure S6.** Forest plot of GLP-1 receptor agonists versus comparators on the risk of Pulmonary fibrosis. GLP-1RAs: GLP-1 receptor agonists, RR: risk ratios, CI: confidence Interval. **Figure S7.** Forest plot of GLP-1 receptor agonists versus comparators on the risk of Dyspnoea. GLP-1RAs: GLP-1 receptor agonists, RR: risk ratios, CI: confidence Interval. **Figure S8.** Forest plot of GLP-1 receptor agonists versus comparators on the risk of Acute respiratory failure. GLP-1RAs: GLP-1 receptor agonists, RR: risk ratios, CI: confidence Interval. **Figure S9.** Forest plot of GLP-1 receptor agonists versus comparators on the risk of Pleural effusion. GLP-1RAs: GLP-1 receptor agonists, RR: risk ratios, CI: confidence Interval. **Figure S10.** Forest plot of GLP-1 receptor agonists versus comparators on the risk of Asthma. GLP-1RAs: GLP-1 receptor agonists, RR: risk ratios, CI: confidence Interval. **Figure S11.** Forest plot of GLP-1 receptor agonists versus comparators on the risk of COPD. GLP-1RAs: GLP-1 receptor agonists, RR: risk ratios, CI: confidence Interval. **Figure S12.** Forest plot of GLP-1 receptor agonists versus comparators on the risk of Sleep apnoea syndrome. GLP-1RAs: GLP-1 receptor agonists, RR: risk ratios, CI: confidence Interval. **Figure S13.** Forest plot of GLP-1 receptor agonists versus comparators on the risk of Pulmonary embolism. GLP-1RAs: GLP-1 receptor agonists, RR: risk ratios, CI: confidence Interval. **Figure S14.** Forest plot of GLP-1 receptor agonists versus comparators on the risk of Pulmonary hypertension. GLP-1RAs: GLP-1 receptor agonists, RR: risk ratios, CI: confidence Interval. **Figure S15.** Subgroup analyses (indication) of the effects of GLP-1 receptor agonists on the risk of overall respiratory disease. **Figure S16.** Subgroup analyses (control type) of the effects of GLP-1 receptor agonists on the risk of overall respiratory disease. **Figure S17.** Subgroup analyses (duration) of the effects of GLP-1 receptor agonists on the risk of overall respiratory disease. **Figure S18.** Forest plot of Albiglutide versus comparators on the risk of overall Respiratory diseases. GLP-1RAs, GLP-1 receptor agonists; RR, risk ratios; CI, confidence interval. **Figure S19.** Forest plot of Dulaglutide versus comparators on the risk of overall Respiratory diseases. GLP-1RAs, GLP-1 receptor agonists; RR, risk ratios; CI, confidence interval. **Figure S20.** Forest plot of Efpeglenatide versus comparators on the risk of overall Respiratory diseases. GLP-1RAs, GLP-1 receptor agonists; RR, risk ratios; CI, confidence interval. **Figure S21.** Forest plot of Exenatide versus comparators on the risk of overall Respiratory diseases. GLP-1RAs, GLP-1 receptor agonists; RR, risk ratios; CI, confidence interval. **Figure S22.** Forest plot of Liraglutide versus comparators on the risk of overall Respiratory diseases. GLP-1RAs, GLP-1 receptor agonists; RR, risk ratios; CI, confidence interval. **Figure S23.** Forest plot of Lixisenatide versus comparators on the risk of overall Respiratory diseases. GLP-1RAs, GLP-1 receptor agonists; RR, risk ratios; CI, confidence interval. **Figure S24.** Forest plot of Semaglutide versus comparators on the risk of overall Respiratory diseases. GLP-1RAs, GLP-1 receptor agonists; RR, risk ratios; CI, confidence interval.

## Data Availability

All data generated or analysed during this study are included in this published article (and its supplementary information files).

## Abbreviations

GLP-1 RAs: Glucagon-like peptide-1 receptor agonists
T2DM: Type 2 diabetes mellitus
COPD: Chronic obstructive pulmonary disease
BMI: Body mass index
HbA1c: Glycated hemoglobin
RR: Relative risk
CI: Confidence interval
RCT: Randomized controlled trial
CVOT: Cardiovascular outcome trial
PRISMA: Preferred reporting items for systematic reviews and meta-analyses
