# Supplementary material for: The relationship between the use of GLP-1 receptor agonists and the incidence of respiratory illness: a meta-analysis of randomized controlled trials
Source: Diabetol Metab Syndr. 2023 Jul 26;15:164. doi: 10.1186/s13098-023-01118-6 (PMC10369734; doi:10.1186/s13098-023-01118-6)
Supplement: Supplementary file 1 — Additional file 1: Figure S1. Risk of bias summary. Figure S2. Funnel plot and Egger’s test for the comparison of the incidence of overall Respiratory diseases with the use of GLP-1 receptor agonists versus placebo or other antidiabetic treatments. Figure S3. Forest plot of GLP-1 receptor agonists versus comparators on the risk of Pulmonary edema. GLP-1RAs: GLP-1 receptor agonists, RR: risk ratios, CI: confidence Interval. Figure S4. Forest plot of GLP-1 receptor agonists versus comparators on the risk of Bronchitis. GLP-1RAs: GLP-1 receptor agonists, RR: risk ratios, CI: confidence Interval. Figure S5. Forest plot of GLP-1 receptor agonists versus comparators on the risk of pneumonia. GLP-1RAs: GLP-1 receptor agonists, RR: risk ratios, CI: confidence Interval. Figure S6. Forest plot of GLP-1 receptor agonists versus comparators on the risk of Pulmonary fibrosis. GLP-1RAs: GLP-1 receptor agonists, RR: risk ratios, CI: confidence Interval. Figure S7. Forest plot of GLP-1 receptor agonists versus comparators on the risk of Dyspnoea. GLP-1RAs: GLP-1 receptor agonists, RR: risk ratios, CI: confidence Interval. Figure S8. Forest plot of GLP-1 receptor agonists versus comparators on the risk of Acute respiratory failure. GLP-1RAs: GLP-1 receptor agonists, RR: risk ratios, CI: confidence Interval. Figure S9. Forest plot of GLP-1 receptor agonists versus comparators on the risk of Pleural effusion. GLP-1RAs: GLP-1 receptor agonists, RR: risk ratios, CI: confidence Interval. Figure S10. Forest plot of GLP-1 receptor agonists versus comparators on the risk of Asthma. GLP-1RAs: GLP-1 receptor agonists, RR: risk ratios, CI: confidence Interval. Figure S11. Forest plot of GLP-1 receptor agonists versus comparators on the risk of COPD. GLP-1RAs: GLP-1 receptor agonists, RR: risk ratios, CI: confidence Interval. Figure S12. Forest plot of GLP-1 receptor agonists versus comparators on the risk of Sleep apnoea syndrome. GLP-1RAs: GLP-1 receptor agonists, RR: risk ratios, CI: confide [file 13098_2023_1118_MOESM1_ESM.docx]

**Appendix**

**Search strategy**

**PubMed**

((((("Diabetes Mellitus, Type 2"[Mesh]) OR (((((((((((((((((Diabetes Mellitus, Adult-Onset[Title/Abstract]) OR (Diabetes Mellitus, Ketosis-Resistant[Title/Abstract])) OR (Diabetes Mellitus, Maturity-Onset[Title/Abstract])) OR (Diabetes Mellitus, Non Insulin Dependent[Title/Abstract]))OR (Diabetes Mellitus, Non-Insulin-Dependent[Title/Abstract])) OR (Diabetes Mellitus, Noninsulin Dependent[Title/Abstract])) OR (Diabetes Mellitus, Noninsulin-Dependent[Title/Abstract])) OR (Diabetes Mellitus, Slow-Onset[Title/Abstract])) OR (Diabetes Mellitus, Stable[Title/Abstract])) OR (Diabetes Mellitus, Type II[Title/Abstract])) OR (MODY[Title/Abstract])) OR (Maturity-Onset Diabetes[Title/Abstract])) OR (Maturity-Onset Diabetes Mellitus[Title/Abstract])) OR (NIDDM[Title/Abstract])) OR (Noninsulin-Dependent Diabetes Mellitus[Title/Abstract])) OR (Type 2 Diabetes[Title/Abstract])) OR (Type 2 Diabetes Mellitus[Title/Abstract]))) OR (("Obesity"[Mesh]) OR (Obese[Title/Abstract]))) OR ("Overweight"[Mesh])) AND (((("Glucagon-Like Peptide 1"[Mesh]) OR ((((Glucagon Like Peptide 1[Title/Abstract]) OR (GLP-1[Title/Abstract])) OR (GLP 1[Title/Abstract])) OR (Glucagon-Like Peptide-1[Title/Abstract]))) AND (((receptor agonist) OR (receptor agonists)) OR (RA))) OR ((((((albiglutide) OR (dulaglutide)) OR (exenatide)) OR (liraglutide)) OR (lixisenatide)) OR (semaglutide)))) AND (randomized controlled trial[Publication Type] OR randomized[Title/Abstract] OR placebo[Title/Abstract])

**Embase**

| **No.** | **Query** | **Results** | **Date** |
| --- | --- | --- | --- |
| #1 | ('diabetes'/exp OR diabetes) AND mellitus, AND type AND ('2'/exp OR 2) | 395193 | 24-Dec-22 |
| #2 | 'diabetes mellitus, adult-onset':ab,ti OR 'diabetes mellitus, ketosis-resistant':ab,ti OR 'diabetes mellitus, maturity-onset':ab,ti OR 'diabetes mellitus, non insulin dependent':ab,ti OR 'diabetes mellitus, non-insulin-dependent':ab,ti OR 'diabetes mellitus, noninsulin dependent':ab,ti OR 'diabetes mellitus, noninsulin-dependent':ab,ti OR 'diabetes mellitus, slow-onset':ab,ti OR 'diabetes mellitus, stable':ab,ti OR 'diabetes mellitus, type ii':ab,ti OR 'mody':ab,ti OR 'maturity-onset diabetes':ab,ti OR 'maturity-onset diabetes mellitus':ab,ti OR 'niddm':ab,ti OR 'noninsulin-dependent diabetes mellitus':ab,ti OR 'type 2 diabetes':ab,ti OR 'type 2 diabetes mellitus':ab,ti | 247285 | 24-Dec-22 |
| #3 | #1 OR #2 | 421122 | 24-Dec-22 |
| #4 | obesity | 704856 | 24-Dec-22 |
| #5 | 'obese':ab,ti | 228414 | 24-Dec-22 |
| #6 | #4 OR #5 | 737379 | 24-Dec-22 |
| #7 | overweight | 128280 | 24-Dec-22 |
| #8 | #3 OR #6 OR #7 | 1074234 | 24-Dec-22 |
| #9 | 'glucagon like' AND peptide AND 1 | 39802 | 24-Dec-22 |
| #10 | 'glucagon like peptide 1':ab,ti OR 'glp-1':ab,ti OR 'glp 1':ab,ti OR 'glucagon-like peptide-1':ab,ti | 28278 | 24-Dec-22 |
| #11 | #9 OR #10 | 41460 | 24-Dec-22 |
| #12 | 'receptor agonist':ab,ti OR 'receptor agonists':ab,ti OR 'ra':ab,ti | 218409 | 24-Dec-22 |
| #13 | #11 AND #12 | 8310 | 24-Dec-22 |
| #14 | 'albiglutide':ab,ti OR 'dulaglutide':ab,ti OR 'exenatide':ab,ti OR 'liraglutide':ab,ti OR 'lixisenatide':ab,ti OR 'semaglutide':ab,ti | 11747 | 24-Dec-22 |
| #15 | #13 OR #14 | 16195 | 24-Dec-22 |
| #16 | 'randomized controlled trial':ab,ti OR 'randomized':ab,ti OR 'placebo':ab,ti OR 'rct':ab,ti | 1107747 | 24-Dec-22 |
| #17 | #8 AND #15 AND #16 | 3861 | 24-Dec-22 |


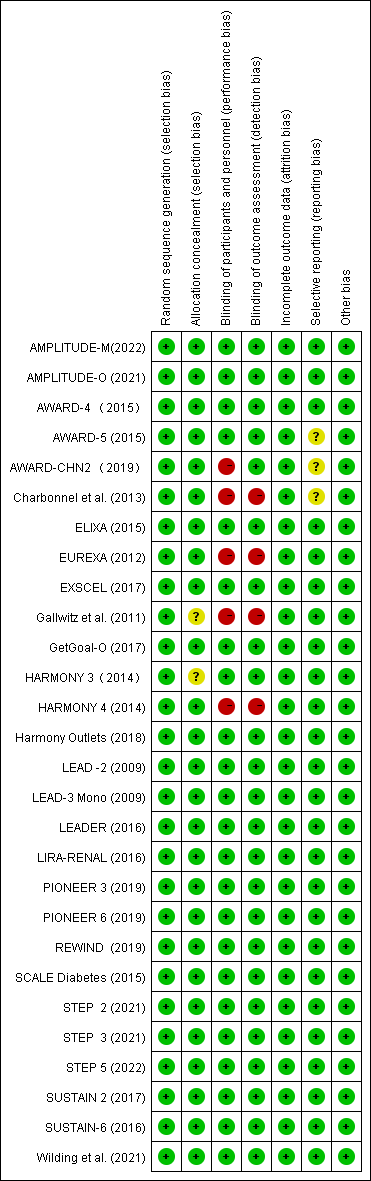


**FIGURE S1.** Risk of bias summary

**
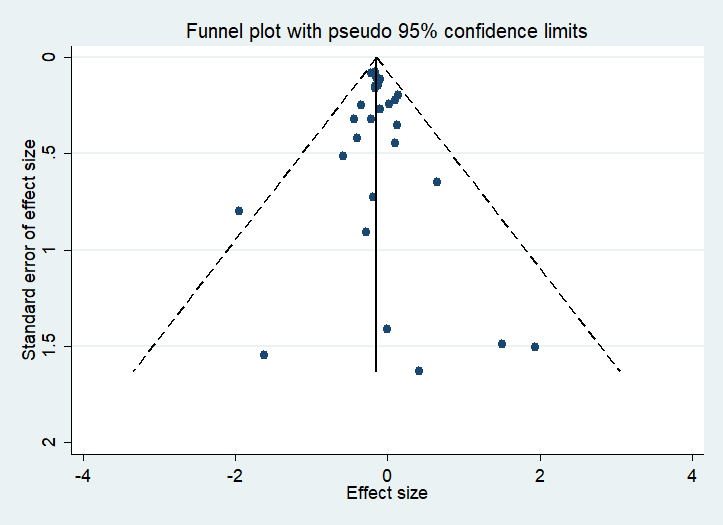
**

**FIGURE S2**. Funnel plot and Egger’s test for the comparison of the incidence of overall Respiratory diseases with the use of GLP-1 receptor agonists versus placebo or other antidiabetic treatments


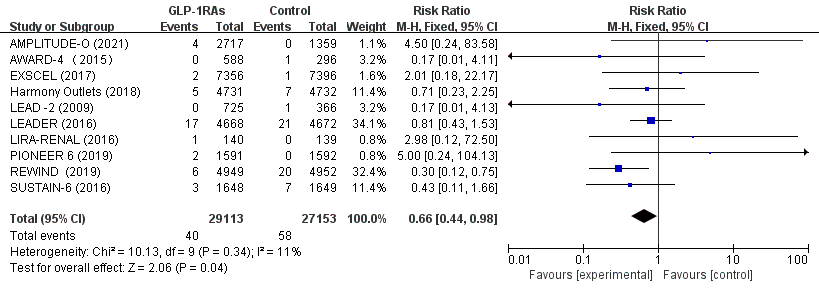


**FIGURE S3.** Forest plot of GLP-1 receptor agonists versus comparators on the risk of

Pulmonary edema. GLP-1RAs: GLP-1 receptor agonists, RR: risk ratios, CI: confidence Interval


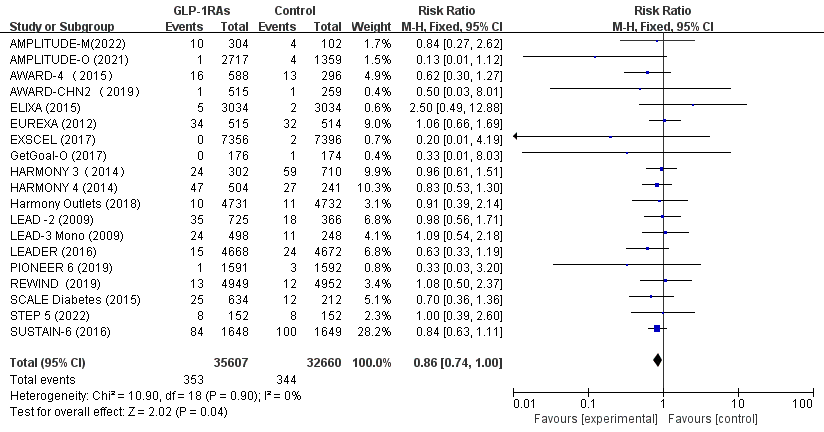


**FIGURE S4.** Forest plot of GLP-1 receptor agonists versus comparators on the risk of

Bronchitis. GLP-1RAs: GLP-1 receptor agonists, RR: risk ratios, CI: confidence Interval


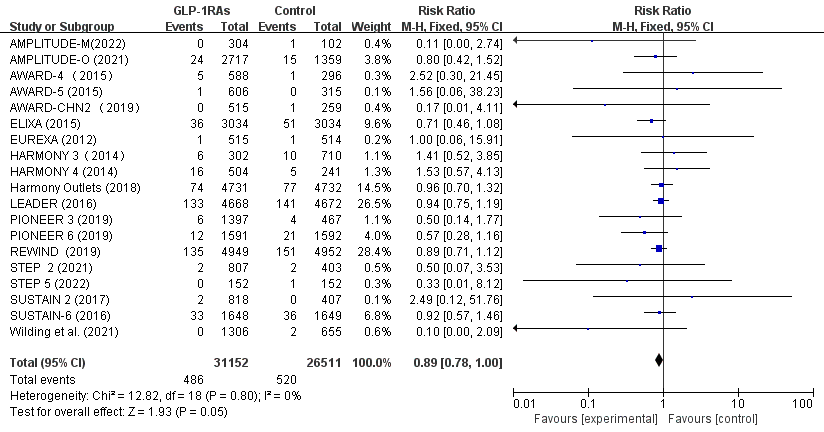


**FIGURE S5**. Forest plot of GLP-1 receptor agonists versus comparators on the risk of

pneumonia. GLP-1RAs: GLP-1 receptor agonists, RR: risk ratios, CI: confidence Interval


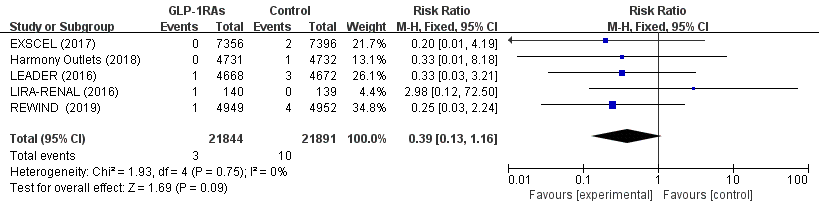


**FIGURE S6**. Forest plot of GLP-1 receptor agonists versus comparators on the risk of

Pulmonary fibrosis. GLP-1RAs: GLP-1 receptor agonists, RR: risk ratios, CI: confidence Interval


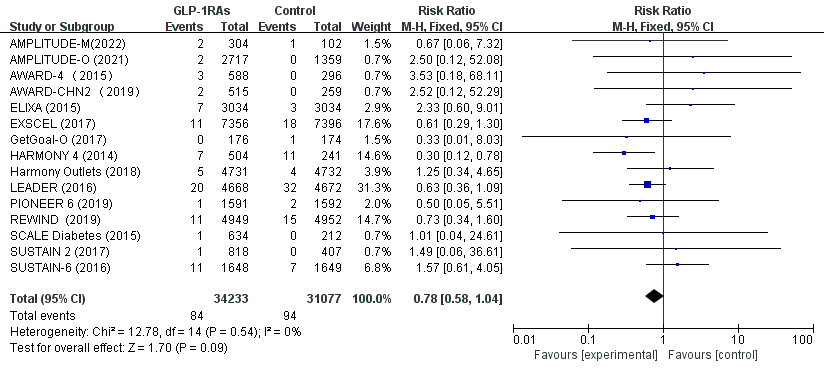


**FIGURE S7**. Forest plot of GLP-1 receptor agonists versus comparators on the risk of

Dyspnoea. GLP-1RAs: GLP-1 receptor agonists, RR: risk ratios, CI: confidence Interval


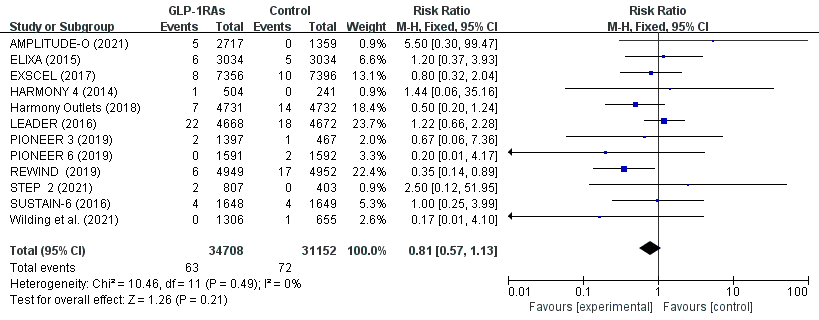


**FIGURE S8**. Forest plot of GLP-1 receptor agonists versus comparators on the risk of

Acute respiratory failure. GLP-1RAs: GLP-1 receptor agonists, RR: risk ratios, CI: confidence Interval


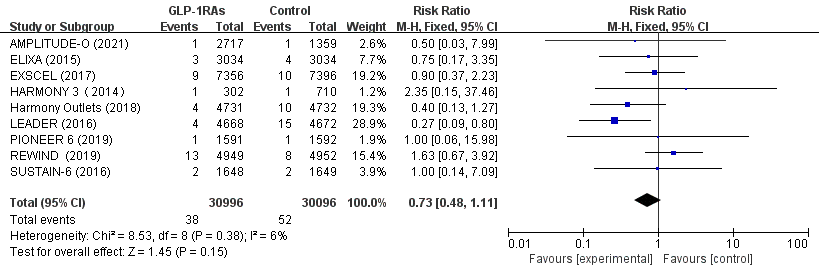


**FIGURE S9**. Forest plot of GLP-1 receptor agonists versus comparators on the risk of

Pleural effusion. GLP-1RAs: GLP-1 receptor agonists, RR: risk ratios, CI: confidence

Interval


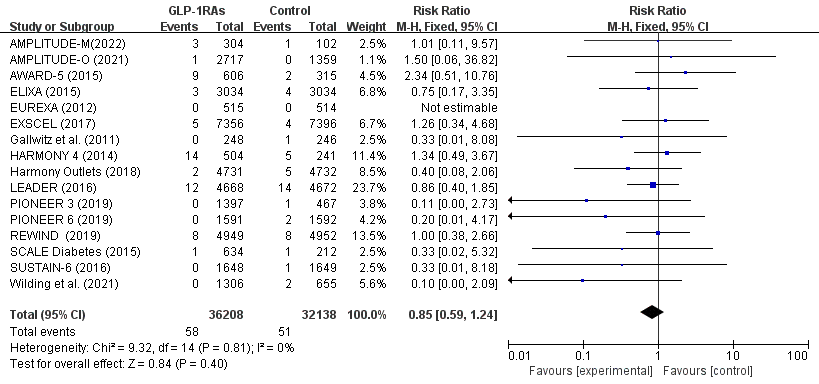


**FIGURE S10**. Forest plot of GLP-1 receptor agonists versus comparators on the risk of Asthma. GLP-1RAs: GLP-1 receptor agonists, RR: risk ratios, CI: confidence

Interval


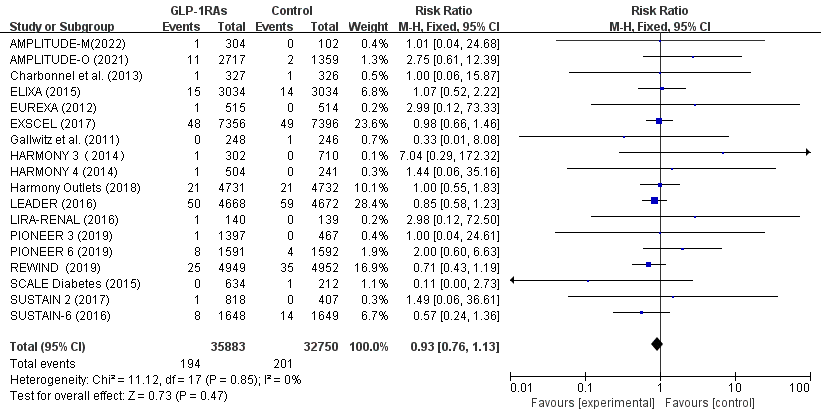


**FIGURE S11**. Forest plot of GLP-1 receptor agonists versus comparators on the risk of COPD. GLP-1RAs: GLP-1 receptor agonists, RR: risk ratios, CI: confidence

Interval


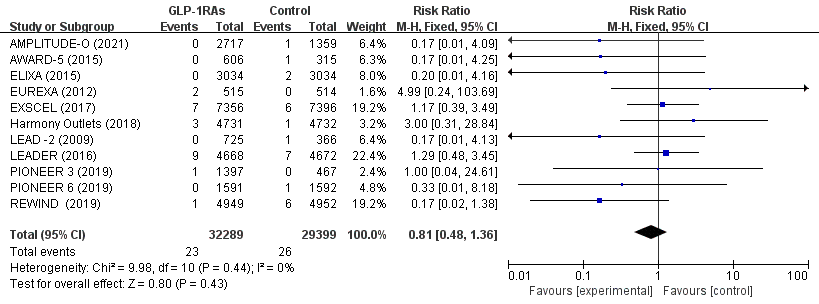


**FIGURE S12**. Forest plot of GLP-1 receptor agonists versus comparators on the risk of Sleep apnoea syndrome. GLP-1RAs: GLP-1 receptor agonists, RR: risk ratios, CI: confidence Interval


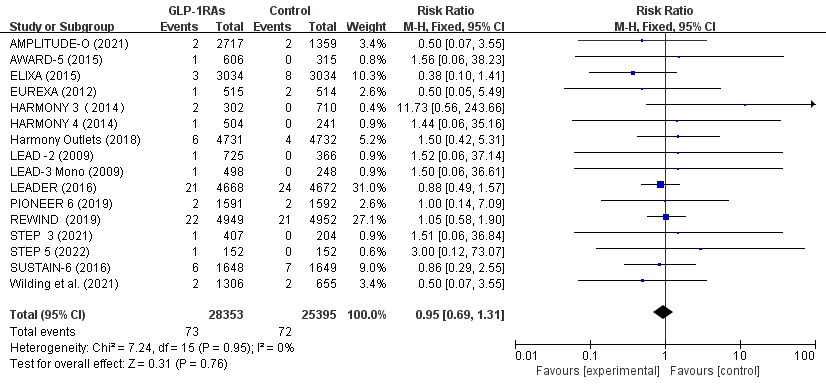


**FIGURE S13**. Forest plot of GLP-1 receptor agonists versus comparators on the risk of Pulmonary embolism. GLP-1RAs: GLP-1 receptor agonists, RR: risk ratios, CI: confidence Interval


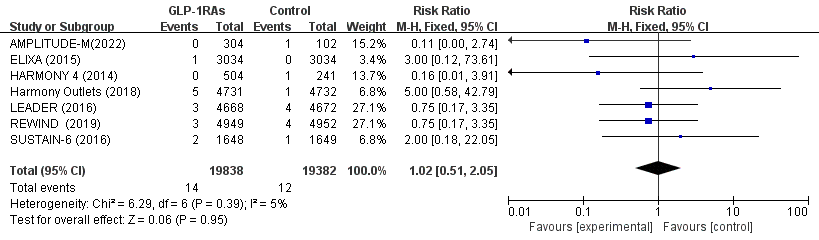


**FIGURE S14**. Forest plot of GLP-1 receptor agonists versus comparators on the risk of Pulmonary hypertension. GLP-1RAs: GLP-1 receptor agonists, RR: risk ratios, CI: confidence Interval


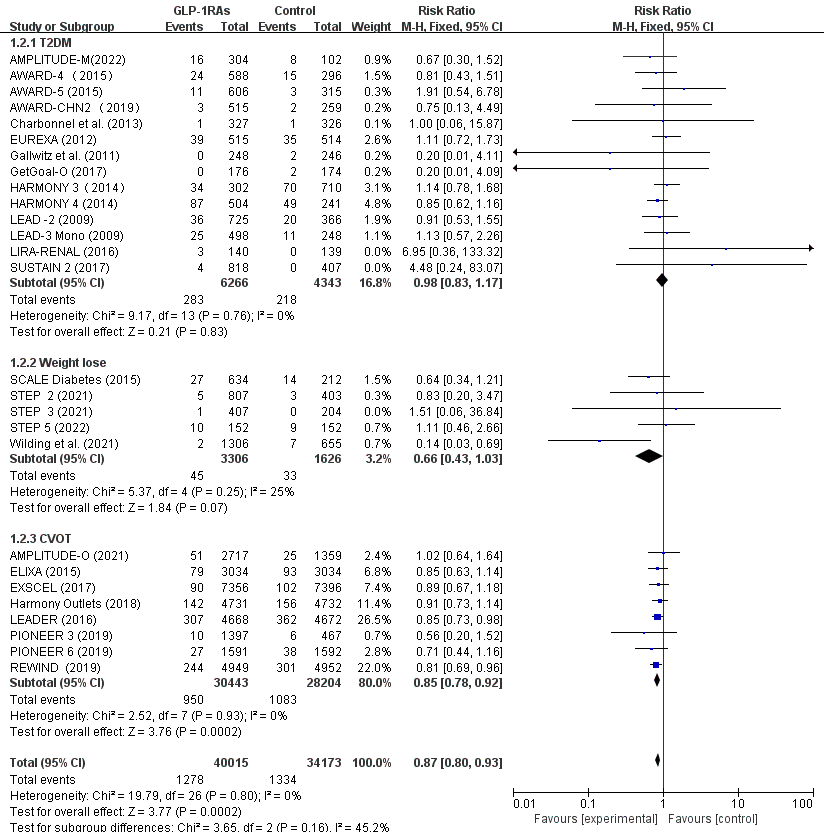


**FIGURE S15**. Subgroup analyses (indication) of the effects of GLP-1 receptor agonists on the risk of overall respiratory disease.


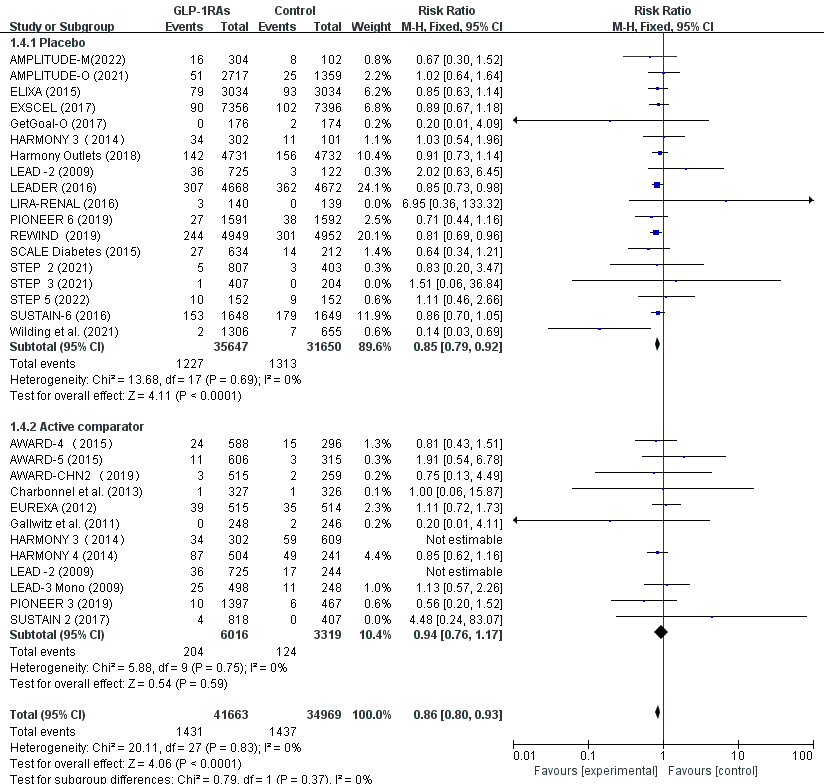


**FIGURE S16**. Subgroup analyses (control type) of the effects of GLP-1 receptor agonists on the risk of overall respiratory disease.


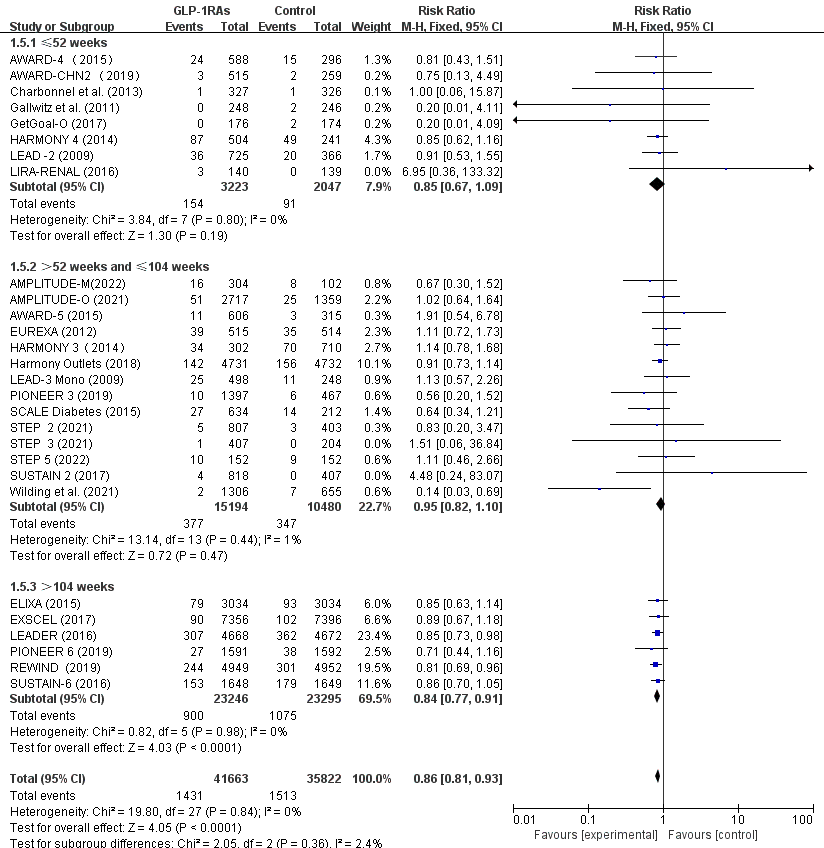


**FIGURE S17**. Subgroup analyses (duration) of the effects of GLP-1 receptor agonists on the risk of overall respiratory disease.


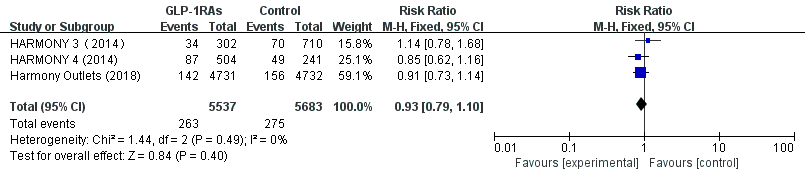


**FIGURE S18**. Forest plot of Albiglutide versus comparators on the risk of overall Respiratory diseases. GLP-1RAs, GLP-1 receptor agonists; RR, risk ratios; CI, confidence interval.


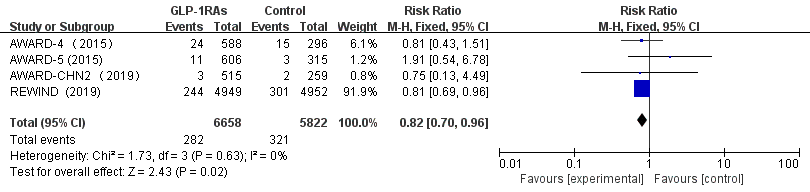


**FIGURE S19**. Forest plot of Dulaglutide versus comparators on the risk of overall Respiratory diseases. GLP-1RAs, GLP-1 receptor agonists; RR, risk ratios; CI, confidence interval.


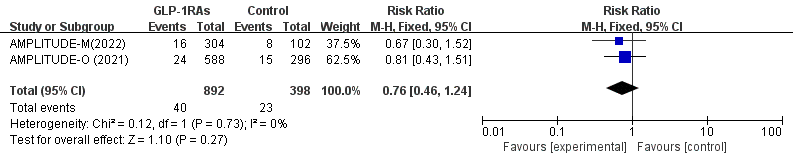


**FIGURE S20**. Forest plot of Efpeglenatide versus comparators on the risk of overall Respiratory diseases. GLP-1RAs, GLP-1 receptor agonists; RR, risk ratios; CI, confidence interval.


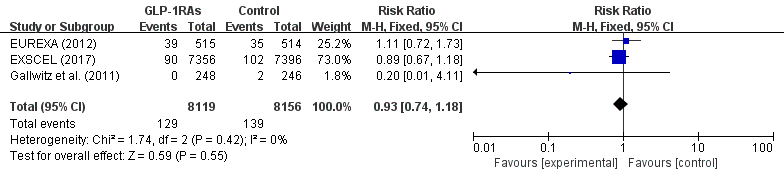


**FIGURE S21**. Forest plot of Exenatide versus comparators on the risk of overall Respiratory diseases. GLP-1RAs, GLP-1 receptor agonists; RR, risk ratios; CI, confidence interval.


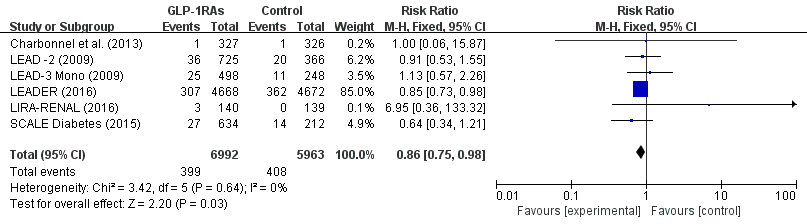


**FIGURE S22**. Forest plot of Liraglutide versus comparators on the risk of overall Respiratory diseases. GLP-1RAs, GLP-1 receptor agonists; RR, risk ratios; CI, confidence interval.


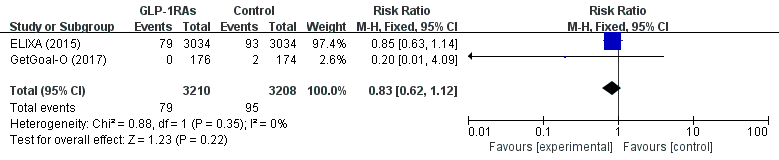


**FIGURE S23**. Forest plot of Lixisenatide versus comparators on the risk of overall Respiratory diseases. GLP-1RAs, GLP-1 receptor agonists; RR, risk ratios; CI, confidence interval.


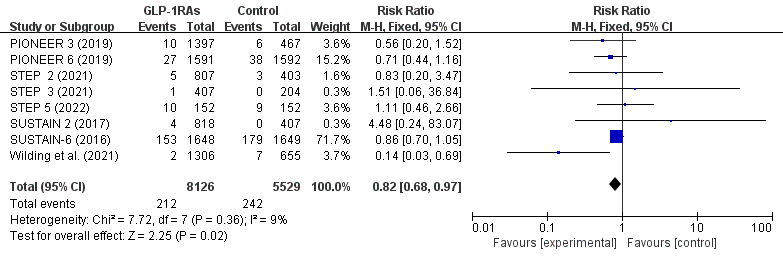


**FIGURE S24**. Forest plot of Semaglutide versus comparators on the risk of overall Respiratory diseases. GLP-1RAs, GLP-1 receptor agonists; RR, risk ratios; CI, confidence interval.
